# Impulsivity among Senior Nursing Students: The role of Emotional Intelligence and clinical performance

**DOI:** 10.17533/udea.iee.v43n2e08

**Published:** 2025-07-25

**Authors:** Anjali Rathee, Priyanshi Dixit, Ankit Raj, Jyoti Bala, Devendra Singh, Lalit Kumar

**Affiliations:** 1 Department of Nursing Services, All India Institute of Medical Sciences, New Delhi, India. Email: anjaliratheee00@gmail.com. https://orcid.org/0009-0004-0460-4917 All India Institute Of Medical Sciences Department of Nursing Services All India Institute of Medical Sciences New Delhi India anjaliratheee00@gmail.com; 2 College of Nursing, Vardhman Mahavir Medical College, Safdarjung Hospital, New Delhi, India. Email: priyanshidixit17@gmail.com. Corresponding author. https://orcid.org/0000-0001-5130-4037 Vardhman Mahavir Medical College College of Nursing Vardhman Mahavir Medical College Safdarjung Hospital New Delhi India priyanshidixit17@gmail.com; 3 Faculty of Nursing. Email: ankitraj92srivastava@gmail.com. https://orcid.org/0009-0001-8418-1114 Uttar Pradesh University of Medical Science Faculty of Nursing India ankitraj92srivastava@gmail.com; 4 Faculty of Nursing. Email: jb1849@gmail.com. https://orcid.org/0000-0003-1732-0636 Uttar Pradesh University of Medical Science Faculty of Nursing India jb1849@gmail.com; 5 Faculty of Nursing. Email: devendrasingh265@gmail.com. https://orcid.org/0009-0001-3191-250X Uttar Pradesh University of Medical Science Faculty of Nursing India devendrasingh265@gmail.com; 6 Faculty of Nursing. Email: lalitk351@gmail.com. https://orcid.org/0009-0008-3316-3216 Uttar Pradesh University of Medical Science Faculty of Nursing India lalitk351@gmail.com; 7 Uttar Pradesh University of Medical Science, Saifai, Uttar Pradesh, India. Uttar Pradesh University of Medical Science Uttar Pradesh University of Medical Science Saifai Uttar Pradesh India

**Keywords:** emotional intelligence, impulsivity, student, nurses, clinical performance., inteligencia emocional, impulsividad, estudiantes enfermeras, desempeño clínico., inteligência emocional, impulsividade, estudantes de enfermagem, desempenho clínico.

## Abstract

**Objective.:**

This study examined the relationship between impulsivity, emotional intelligence, and clinical performance.

**Methods.:**

Through a total enumerative sampling technique, this correlational study involved 229 pre-final and final-year nursing students from Northern India. Data were collected using validated questionnaires measuring Emotional Intelligence -EI- (Schutte Self-Report Emotional Intelligence Scale), Barratt Impulsiveness Scale-Brief (BIS-Brief), and clinical competence (Clinical Competence Questionnaire).

**Results.:**

All participants were nursing students, with 62% in their pre-final year; however, only 83.41% expressed interest in pursuing a nursing career. A significant negative correlation of impulsivity with self-awareness (r=-0.54, *p*=0.021) and advanced nursing skills (r=-0.61, *p*=0.031) was found. Among selected socio-demographic variables, age (*p*=0.049) emerged as a significant positive predictor of impulsive behaviour.

**Conclusion.:**

The findings suggest impulsivity is inversely related to self-awareness, a domain of EI, and advanced nursing skills, implying that students with higher self-awareness exhibit better emotional regulation and decision-making in clinical settings.

## Resumen

**Objetivo.:**

Investigar la relación entre impulsividad, inteligencia emocional y rendimiento clínico.

**Métodos.:**

En este estudio correlacional participaron 229 estudiantes de enfermería de último y penúltimo año de una universidad del norte de la India, los cuales fueron seleccionados mediante una técnica de muestreo enumerativo total. Los datos se recogieron utilizando cuestionarios validados que medían la Inteligencia Emocional -EI- (Schutte Self-Report Emotional Intelligence Scale), Barratt Impulsiveness Scale-Brief (BIS Brief), y la competencia clínica (Clinical Competence Questionnaire).

**Resultados.:**

Más de la mitad de las participantes (62%) cursaban el último año de la carrera de enfermería y el 83.41% manifestaron interés por la profesión. Se encontró una correlación negativa significativa de la impulsividad con la autoconciencia (r=-0.54, *p*=0.021) y las habilidades avanzadas de enfermería (r=-0.61, *p*=0.031). Entre las variables sociodemográficas seleccionadas, la edad (p=0.049) resultó ser un predictor positivo significativo del comportamiento impulsivo**.**

**Conclusión.:**

Los hallazgos sugieren que la impulsividad está inversamente relacionada con la autoconciencia -un dominio de la IE-, y las habilidades avanzadas de enfermería, lo que implica que los estudiantes con mayor autoconciencia muestran una mejor regulación emocional y tienen adecuadas tomas de decisiones en entornos clínicos.

## Resumo

**Objetivo.:**

Investigar a relação entre impulsividade, inteligência emocional e desempenho clínico.

**Métodos.:**

Este estudo correlacional envolveu 229 estudantes de enfermagem do último e penúltimo ano de uma universidade no norte da Índia. Eles foram selecionados por meio de uma técnica de amostragem enumerativa total. Os dados foram coletados por meio de questionários validados que mensuram a Inteligência Emocional (IE) (*Schutte Self-Report Emotional Intelligence Scale), Barratt Impulsiveness Scale-Brief* (BIS *Brief*) e competência clínica (*Clinical Competence Questionnaire*).

**Resultados.:**

Mais da metade dos participantes (62%) estavam no último ano do curso de enfermagem. Foi encontrada uma correlação negativa significativa entre impulsividade e autoconsciência (*r*=-0.54, *p*<0.05) e habilidades avançadas de enfermagem (*r*=-0.61, *p*<0.05). Entre as variáveis sociodemográficas selecionadas, a idade (*p<*0.05) foi considerada um preditor positivo significativo do comportamento impulsivo.

**Conclusão.:**

Os resultados sugerem que a impulsividade está inversamente relacionada à autoconsciência - um domínio da IE - e às habilidades avançadas de enfermagem, o que implica que os alunos com maior autoconsciência apresentam melhor regulação emocional e tomada de decisão adequada em ambientes clínicos.

## Introduction

Emotional intelligence (EI), as defined by Salovey and Mayer,^1^ is a form of social intelligence that involves the ability to recognise and understand both one's own and others' emotions, distinguish between them, and use this awareness to guide thoughts and actions. Nurses and nurse educators must understand the idea of EI and be familiar with the theories and research that underpin it. Nursing is considered a high-pressure and demanding profession. Nursing students are expected to play a key role in providing care services soon, requiring them to develop the necessary clinical skills to enhance patient care quality, and make them more competent in clinical areas, so incorporating EI may give an advantage among nursing students. For enhancing clinical performance, students nurse requires knowledge, skills, attitudes, and abilities, as well as applying technical, communication, and clinical reasoning skills to deliver effective care in clinical settings, and this competence develops over time through practice, experience, and quality education.^2^^,^^3^ Clinical performance is vital for professional growth, confidence in the workplace, and ensuring safe and efficient care. Nurses with higher clinical competence are more adept at building empathetic connections with patients and applying their skills effectively in clinical settings. As a result, clinical competence plays a vital role in enhancing care quality, patient satisfaction, and the sustainability of hospitals in today’s competitive healthcare environment.^4^

While some studies from other countries have established a clear link between emotional intelligence and better performance in clinical areas and observed relationships between EI and clinical performance, higher emotionally intelligent student nurses maintained better patient relationships, clinical reasoning, self-awareness, and adaptability^5^ as compared to lower EI students. Whereas other research has found no such correlation between EI and clinical competence. Impulsivity may also act as a significant predictor of performing better in a clinical setting through EI. Low EI is characterised by poor emotional reasoning that can lead to more impulsive reactions to stressful situations. Enhancing emotional clarity (understanding emotions) and emotional regulation (managing emotions) could help mitigate impulsive behaviour.^6^ Higher impulsive behaviour leads to difficulty in inhibiting responses and delaying gratification, often leading to choices based on immediate rewards rather than long-term benefits.^7^ This impulsive behaviour may be because a person acts without prior planning or consideration, responding impulsively to internal or external stimuli and ignoring possible negative consequences.^8^ Major factors for higher impulsive behaviours are if the person (students) doesn’t understand their own or others’ emotional states and responds inappropriately to threats or frustrations, resulting in impulsivity. 

Understanding own as well as others' emotions is one of the crucial components of emotional intelligence. In this way, EI and impulsivity seem to be interconnected. While many studies have explored the relationship between emotional intelligence and the clinical performance of nursing students, none have examined the link between impulsivity, emotional intelligence, and clinical performance. Given the limited research on impulsivity and its association with EI and clinical performance among nursing students, this study aimed to examine the relationship between impulsivity, EI and clinical performance among senior nursing students so that the findings of this study can address the present gaps in the literature. Specifically, this study seeks to address the following research questions: (i) How do impulsivity, EI and clinical performance differ among pre-final and final-year nursing students? and (ii) What are the factors associated with impulsivity among senior nursing students? By examining these factors together, this study sought to provide a more comprehensive understanding of the factors influencing nursing students' impulsivity. So, curriculum developers inculcate measures to enhance EI and decrease impulsivity for better control of emotion during patient care. 

## Methods

Study design and setting. A correlational study using a total enumerative sampling technique was conducted to investigate the role of EI and clinical performance on nursing students' impulsivity. The sample of this study consists of 229 pre-final and final-year bachelor’s nursing students, ≥ 18 years of age, and willing to participate in the study. These specific years were chosen because students have gained sufficient clinical experience at this educational stage. Data were collected from August 2024 to December 2024 from a nursing college in Northern India. 

Data collection tools. This study utilized several data-collection tools. A data sheet was created to elicit information related to socio-demographic variables. Impulsivity among nursing students was assessed using the Barratt Impulsiveness Scale-Brief (BIS-Brief). BIS is an 8-item questionnaire that evaluates general impulsiveness or the absence of future planning and forethought. It is a valid and reliable tool. The estimated Cronbach’s alpha for the scale was 0.78.^9^ Schutte Self-Report Emotional Intelligence Scale (SSREI) with Cronbach’s alpha=0.90 validity (CVI=0.88) was used to measure emotional intelligence. The SSREI is a five-point Likert scale ranging from 1 (strongly disagree) to 5 (strongly agree) and includes 33 items. EI was measured as the sum of the scores for all 33 items, with a score range of 33-165. A score below 124 suggested a low EI level, and 124 or higher denoted a high level of EI.^10^ The Clinical Competence Questionnaire (CCQ) was used to evaluate the clinical competency of the nursing students. It consists of 47 items that represent clinical competencies categorised as either nursing professional behaviour (items 1-17), or related to clinical competency (items 17-47). The CCQ uses a 5-point Likert scale, ranging from 1 (do not have a clue) to 5 (known in theory, competent in practice without supervision). The total score ranges from 47 to 235, with higher scores indicating good clinical competence. The Cronbach’s alpha for the entire 47-item Clinical Competence Questionnaire was 0.98.^11^

Ethical considerations. Before participation, the participants were informed of the aim and purpose of the study. Informed written consent was obtained before data collection. Confidentiality and anonymity were maintained. The Institutional Ethics Committee of the Uttar Pradesh University of Medical Sciences, with reference number: 167/Cert./IEC/UPUMS/2023-24, dated 11/03/2024. Written informed consent was obtained from all eligible participants.

Statistical analysis. We conducted data analysis and interpretation to reduce, organise, and give meaning to the data. Statistical analyses were carried out using SPSS software (version 26.0; IBM Corp., Armonk, N.Y., USA). Both descriptive and inferential statistics were employed to analyse the data from the current study. Descriptive statistics, including percentages, means, and standard deviations, were calculated. A chi-square test was used for comparative analysis between the poor and good EI groups. Independent t-tests were conducted to examine differences in mean EI, clinical competence levels, and impulsivity across academic years. Pearson correlation was applied to evaluate the correlation between impulsivity and EI with clinical competence. One-way ANOVA and independent t-tests were used to find the association of socio-demographic variables with impulsivity. Multivariate linear regression analyses were performed to identify predictors of impulsiveness. All statistical tests were two-tailed, with statistical significance set at *p*<0.05.

## Results

**Socio-demographic profile of participants:**

The study included 229 nursing students (response rate: 81.84%), the majority of whom were aged 22-23 years (65.50%) and unmarried (97.81%). Most participants (62%) were in the pre-final year of their nursing programs. The majority, 83.41%, expressed an interest in nursing and were in favour of accepting the nursing profession as a job in future (Table 1). Among the whole participants, 16.59 % did not like nursing as they were in not in favour of doing a nursing job in future. 

**Table 1:** Socio-demographic Profile of 229 Nursing Students

Variables	Categories	***n* (%)**
Age (years)	19-21	53 (23.14)
	22-23	150 (65.50)
	24-25	26 (11.36)
Religion	Hindu	208 (90.83)
	Other	21 (9.17)
Academic Year	Third	142 (62)
	Fourth	87 (38)
	Unmarried	224 (97.81)
	Married	5 (2.19)
Any Psychiatric illness	No	214 (93.45)
	Yes	15 (6.55)
Interested in Nursing	Yes	191 (83.41)
	No	38 (16.59)

Emotional Intelligence, Clinical Competence and Impulsivity Scores by Academic Years: 

Table 2 shows the mean score differences in study variables between third-year and fourth-year students. Fourth-year students scored significantly higher than third-year students in all EI and clinical competence domains, except for self-control and nursing professional behaviour.

**Table 2:** Impulsivity, Emotional Intelligence and Clinical Competence Scores by Academic Year (*n*=229)

Variable	3^rd^ year Mean (SD)	4^th^ year Mean (SD)	t-value	***p*-value**
BIS	15.13 (3.59)	15.88 (3.71)	-1.53	0.122
EI- Motivation	25.55 (4.06)	26.76 (3.12)	-2.38	0.015
EI- Self-awareness	29.28 (3.53)	30.51 (2.83)	-2.75	0.016
EI- Self-control	25.60 (3.56)	26.40 (3.12)	-1.72	0.081
EI- Social Consciousness	22.05 (3.24)	23.10 (2.41)	-2.60	0.010
EI- Social Skills	17.82 (2.65)	18.71 (2.09)	-2.66	0.015
Over EI	120.32 (11.86)	125.49 (11.43)	-2.78	0.069
CCQ-Nursing Professional Behaviours	57.01 (14.07)	60.19 (12.01)	-1.76	0.081
CCQ-General Performance	44.53 (11.51)	48.00 (9.01)	-2.39	0.021
CCQ-Core Nursing Skills	47.62 (12.39)	51.95 (9.30)	-2.81	0.016
CCQ-Advanced Nursing Skills	22.25 (5.46)	24.41 (4.59)	-3.09	0.016
Overall CCQ	175.27 (41.39)	188.79 (32.46)	-2.59	0.012
Abbreviations: EI- Emotional Intelligence; SSEIT- Schutte Self Report Emotional Intelligence Test; CCQ- Clinical Competence Questionnaire; BIS- Barratt Impulsiveness Scale

Participants were split into two groups based on their EI scores (below 124 and 124 or higher). (Supplementary Table 1) These groups had no significant differences regarding demographics such as age, religion, marital status, or psychiatric history. However, differences were found in academic year and interest in nursing, suggesting that EI might be related to a student's academic progression and passion for the nursing field. 

**Supplementary Table 1:** Comparison of Clinical and Demographic Profiles between the Participants with Poor and Good Emotional Intelligence Scores. (*n*=229)

Variables	Categories	Poor EI < 124 (*n* = 116) *n* (%)	Good EI ≥ 124 (*n* = 113) *n* (%)	***p*-value**
Age (years)	19-21	27 (50.94)	26 (49.06)	0.891
	22-23	77 (51.33)	73 (48.67)	
	24-25	12 (46.15)	14 (53.85)	
Religion	Hindu	105 (50.48)	103 (49.52)	0.214
	Other	11 (52.38)	10 (47.62)	
Academic Year	Third	85 (59.86)	57 (40.14)	0.015
	Fourth	31 (35.63)	56 (64.37)	
Marital Status	Unmarried	114 (50.89)	110 (49.11)	0.681
	Married	2 (40)	3 (60)	
Any Psychiatric illness	No	105 (49.07)	109 (50.93)	0.076
	Yes	11 (73.33)	4 (26.67)	
Interested in Nursing	Yes	88 (46.07)	103 (53.93)	0.022
	No	28 (73.68)	10 (26.32)	

Correlation of Impulsivity with Emotional Intelligence and Clinical Competence: 

Table 3 indicates a significant negative correlation of impulsivity with self-awareness (r=-0.54, p≤0.05), meaning less self-aware students tended to be more impulsive. However, no relationship was found between impulsivity and all parameters of clinical competence except advanced nursing skills (r=-0.61, p≤0.05). 

**Table 3:** Correlation of Impulsivity with Emotional Intelligence and Clinical Competence (*n*=229)

	Impulsivity	***p*-value**
EI- Motivation	0.05	0.621
EI- Self-Awareness	-0.54	0.021
EI- Self-control	0.07	0.711
EI- Social Consciousness	0.04	0.073
EI- Social Skills	0.07	0.124
Total-EI	0.08	0.985
CCQ-Nursing Professional Behaviours	0.01	0.176
CCQ-General Performance	0.01	0.548
CCQ-Core Nursing Skills	0.01	0.283
CCQ-Advanced Nursing Skills	-0.61	0.031
Total CCQ	-0.01	0.192

Abbreviations: SSEIT- Schutte Self-Report Emotional Intelligence Test; CCQ- Clinical Competence Questionnaire.

Selected socio-demographic variables were not found to be associated with impulsivity, except age, with p<0.05. (Table 4) Impulsivity was observed to increase with age, meaning older participants were more likely to display impulsive behaviour. 

**Table 4:** Socio-demographic Factors Associated with Impulsivity in Univariate Analyses (*n*=229)

Variables			Impulsivity	
			Mean (SD)		***p*-value**
Age (years)	19-21	14.47 (3.67)	0.049
22-23	15.59 (3.42)	
24-25	16.31 (4.55)	
Religion	Hindu	15.54 (3.69)	0.973
	Other	15.50 (3.07)	
Academic Year	Third	15.13 (3.59)	0.135
	Fourth	15.89 (3.71)	
Marital Status	Unmarried	15.44 (3.66)	0.539
	Married	14.40 (3.01)	
Any Psychiatric illness	No	15.45 (3.53)	0.552
	Yes	14.87 (5.21)	
Interested in Nursing	Yes	15.42 (3.66)	0.931
No	15.37 (3.65)	

Additionally, we conducted a multiple linear regression analysis to identify significant predictors of impulsiveness. The dependent variable (impulsiveness) was regressed on the predicting variables of age, EI, and clinical competence (R²=0.094; adjusted R²=0.021). Age and self-awareness were found to be significant predictors of impulsiveness (p<0.05) (Table 5).

**Table 5:** Multiple Linear Regression Analysis of Factors Associated with Impulsivity

Variables	Unstandardized Coefficients		Standardized Coefficients	T	Sig.	95% Confidence Interval for B		
	B	Std. Error	Beta			Lower Bound	Upper Bound
Age	0.91	0.44	0.14	2.07	0.032	0.04	1.78
Psychiatric illness	-0.92	1.00	-0.06	-0.91	0.362	-2.90	1.06
Interested in Nursing	0.16	0.69	0.01	0.23	0.814	-1.21	1.53
Marital Status	-0.83	1.79	-0.03	-0.46	0.642	-4.37	2.69
Academic Year	0.43	0.53	0.05	0.81	0.415	-0.62	1.50
Religion	-0.54	0.43	-0.08	-1.23	0.212	-1.41	0.32
EI-Motivation	-0.09	0.12	-0.09	-0.74	0.452	-0.33	0.14
EI-Self-Awareness	-0.22	0.11	-0.20	-1.91	0.049	-0.01	0.46
EI-Self-Control	0.06	0.12	0.06	0.53	0.595	-0.17	0.30
EI-Social Consciousness	-0.13	0.14	-0.11	-0.93	0.352	-0.41	0.14
EI-Social Skills	0.02	0.13	0.01	0.19	0.842	-0.24	0.29
Overall EI	0.72	0.21	0.08	1.23	0.753	-0.78	0.21
CCQ-Nursing Professional Behaviours	-0.89	0.48	-3.29	-1.84	0.065	-1.85	0.06
CCQ-General Performance	-0.95	0.53	-2.79	-1.78	0.072	-2.01	0.09
CCQ-Core Nursing Skills	-0.79	0.50	-2.51	-1.58	0.115	-1.79	0.19
CCQ-Advanced Nursing Skills	-1.07	0.49	-1.55	-2.17	0.031	-2.05	-0.10
Overall CCQ	0.88	0.48	9.37	1.80	0.071	-0.08	1.84

Abbreviations: EI- Emotional Intelligence; SSEIT- Schutte Self Report Emotional Intelligence Test; CCQ- Clinical Competence Questionnaire

## Discussion

According to the present study, there was an association between impulsive behaviour among nursing students, and the factors that were influencing impulsivity were age, the self-awareness domain of emotional intelligence, and advanced nursing skills. Findings also suggested that students with higher self-awareness had better self-control with low impulsive behaviour, which ultimately leads to perfection in advanced nursing skills. Concerning academic progression (3^rd^ vs 4^th^) and interest in nursing were positively associated with good EI scores. The results are consistent with previous studies and show that young people who demonstrate low EI levels also tend to be more impulsive.^12^ While one study reported that impulsivity was associated with overall EI, another study^13^ reported that emotional intelligence does not significantly predict emotional behaviour. 

Higher EI abilities, particularly the ability to manage emotions, would allow us to control our impulsivity associated with positive and negative emotional states, manage the tendency toward seeking new and exciting sensations, and reduce our emotional reactivity to rewards.^14^ One study observed a strong negative correlation between EI and impulsiveness, so lack of support and dysfunctional emotional regulation were found as factors for impulsive behaviours.^15^ They said that students' emotional, social, and cognitive competencies and ability to self-regulate are excellent for their success in managing interpersonal relationships, completing daily activities, meeting varying needs and resolving conflict. This study also observed neither a significant difference in impulsivity by gender, nor did impulsiveness change between academic specialisations or years of study. The impulsivity has been related to empathy and risk-taking behaviour in a study^16^ which concluded that medical students had higher empathy and impulsivity when compared to nursing students, and this could lead to harmed patient satisfaction outcomes, so impulsive behaviour negatively associates with clinical performance. 

Emotional maturity, depending on their age, was reported by a study and showed a direct relationship between nursing students’ age (rs = 0.18; *p* < 0.05) and their EI. EI increases with age, thus older people are more socially intelligent and better able to manage their way of feeling and understanding others' feelings and body language too.^17^ In the present study, participants in the age range of 22-23 had higher EI than those aged 19-21, however, we didn’t find a significant association. The same result was also reported by the study.^18^ Coming into the academic year, the present study found that 4th-year students had significantly higher emotional intelligence as compared to 3rd-year students; This finding is in line with a study^19^ that also reported that students, by the end of the eighth (the last semester), had acquired emotional intelligence components such as self-awareness. Oppositely, a study ^20^ showed that 2nd-year students had higher emotional intelligence than senior students. This conflict in findings can be attributed to the fact that seniors usually have higher emotional intelligence, but juniors can also develop higher emotional intelligence by participating in various cultural programmes and curricular activities, so this trait does not come with age or seniority, but with exposing themselves to outdoor activities. A study done by Abdali *et al.*^21^ concluded that there is no association between EI with age and gender, and others ^19^ concluded no association with the gender of the students. A study^22^ observed that EI has no association with gender, education, and interest in nursing, but was associated with satisfaction with the socioeconomic status of the family. 

Concerning to area of interest in nursing study^22^ concluded that those interested in nursing studies had higher EI than those who were not interested, even though this difference was not significant. In contrast with these findings present study was also reported as significant. EI is considered one of the determining factors of people's adjustment, and, therefore, those who have more adjustment abilities have higher emotional intelligence.^23^The positive correlation of emotional intelligence with clinical competency and its components was also reported in the present study, which is in agreement with the study.^24^^,^^25^ Although a study^26^ found a relation between performing tasks with patients (communication abilities) was associated with emotional intelligence. Understanding emotions properly enables better control in mood monitoring, so it can be assumed that Students with high EI managed to stay in positive moods longer and generate positive results even in emergency times.^27^

The Clinical competence of nurses was also assessed, and the results showed a positive relationship between emotional intelligence and clinical performance.^28^^,^^29^ A consistent result was also observed by the present study, but only a significant difference was observed with the sub-domain of clinical competence, i.e. advanced skills. It is important to make sure nursing students are not ignoring their feelings but allowing them to be felt because they hope to be able to manage challenging and strong feelings and positively channel this so they can move about calmly and professionally.^30^ A study^29^measured students' performance in the six dimensions leadership, critical care, education and collaboration, planning, interpersonal relations and professional development, all of which had a significant positive correlation with emotional intelligence, congruent results were also observed in residents’ performance, whereas another study examined no correlation between emotional intelligence and residents' performance^31^ discussed the subscales of EI, it found that social skills had a significant relationship with clinical competence and concluded the importance of emotions in decisions of daily work domains. 

Limitations and future directions: The present study includes several limitations, first, a self-reporting measure. To begin with, self-reporting tools may lead to compromised levels of honesty. Second, a large number of questions and the length of the questionnaire overall may have impacted the reactivity and quality of the data retrieved, and students may have lost interest while filling form, which could lead to bias. Third, all participants were from the government college, which restricts the generalizability of the findings. Only a few demographic variables were examined ultimately. Educational institutions should incorporate structured EI development programs, including self-awareness exercises and emotional regulation techniques, to enhance students’ ability to manage impulsive behaviours effectively, which can improve clinical performance and patient care. Longitudinal studies should be conducted to explore the long-term impact of EI on impulsivity and clinical performance across various demographic and institutional settings. These recommendations aim to strengthen nursing education by promoting emotional intelligence development, which is crucial for clinical competence and effective patient care. The findings of this study indicate the need for further investigation into the interplay between EI, impulsivity, and clinical competence in different nursing education settings.

Implications: Educational institutions should incorporate structured EI development programs, including self-awareness exercises and emotional regulation techniques, to enhance students’ ability to manage impulsive behaviours effectively, which can improve clinical performance and patient care. Developing targeted interventions to improve clinical competence and reduce impulsivity, particularly for students with lower EI scores, focusing on self-awareness, is crucial.

Conclusion: This study examined the relationships among emotional intelligence (EI), clinical competence, and impulsivity in nursing students. The findings revealed that fourth-year students demonstrated significantly higher levels of EI and clinical competence compared to third-year students, suggesting that both emotional and professional skills develop with academic progression. Interestingly, while impulsivity did not significantly differ between academic years, it was negatively associated with self-awareness and advanced nursing skills. Furthermore, age emerged as a positive predictor of impulsivity, indicating that older students tended to display higher levels of impulsive behaviour. Self-awareness, a core component of EI, was a significant negative predictor of impulsivity, underscoring its potential as a protective factor. These insights highlight the importance of fostering emotional intelligence and self-awareness in nursing education to enhance clinical competence and mitigate impulsivity.

Acknowledgements: We express our gratitude to all the study participants for their cooperation and devotion to time during the data collection period.

Availability of data and materials: The data that support the findings of this study are available upon request from the corresponding author. The data are not publicly available because of privacy or ethical restrictions.
